# Network stress: a wiring diagram of whole stress genes

**DOI:** 10.1093/hr/uhaf302

**Published:** 2025-10-29

**Authors:** Yu Wang, Rongling Wu

**Affiliations:** Beijing Key Laboratory of Topological Statistics and Applications for Complex Systems, Beijing Institute of Mathematical Sciences and Applications, Beijing 101408, China; Yau Mathematical Sciences Center, Tsinghua University, Beijing 100084, China; Beijing Key Laboratory of Topological Statistics and Applications for Complex Systems, Beijing Institute of Mathematical Sciences and Applications, Beijing 101408, China; Yau Mathematical Sciences Center, Tsinghua University, Beijing 100084, China; Program in Applied Statistics, Shanghai Institute for Mathematics and Interdisciplinary Sciences, Shanghai 200433, China

## Abstract

Stress sensitivity and tolerance are the consequences of coordinated regulation by multiple genes. Existing genetic tools can identify key genes that mediate metabolic and physiological processes sensing and perceiving stresses. However, it has become increasingly clear that the end-point phenotype of stress response resulting from these intermediate processes involves intricate but well-coordinated networks constituted by a large array of genes. Here, we describe an emerging functional game-graph theory to coalesce all genes from mapping or association studies into genetic interaction networks. These networks enable geneticists to trace, visualize, and interrogate the precise roadmap of how each gene acts and interacts with every other gene to mediate stress response. By shifting reductionist thinking to a holistic, systems-oriented thinking, this theory overcomes a major challenge of elucidating the detailed genetic architecture of stress response.

## Stress response is an ecological evolutionary developmental process

Every organism experiences various forms of stresses in its lifetime. While responding to stress signals, the organism may use two strategies: the first involves triggering transcriptional and metabolic reprogramming, as a means to maintain homeostasis and prevent cellular impairment [1, 2], and the second pertains to active reduction of growth, an attempt to maximize the likelihood of survival [3]. Both these strategies come with trade-offs as evidenced by the fact that stress-tolerant organisms tend to have lower growth rates and production [3, 4]. Current studies in stress biology mainly focus on characterizing the details of molecular, genomic, and biochemical mechanisms that shape an organism’s defense against stresses [5–9]. Yet, little is known about the genetic architecture underlying the trade-offs between stress tolerance and growth reduction, thereby limiting our capacity to engineer stress-resistant and fast-growing genotypes.

Trade-offs of these two response strategies can be explained as an ecological evolutionary developmental (eco-evo-devo) process. The homeostasis strategy conforms to developmental canalization (or robustness), which produces a consistent phenotype from one environment (e.g. stress free) to the next (e.g. stress exposed) to minimize its loss under stress [10]. The growth reduction strategy is relevant to phenotypic plasticity, which allows an organism to change its phenotype to better adapt to a stress [11–17]. The organism becomes adaptive to stress by sensing stress signals early in life, followed by the development of adaptive phenotypes in later stages of life [18, 19]. From this point of view, the end-point phenotype of stress response is the consequence of complex interactions of developmental canalization and phenotypic plasticity with the third eco-evo-devo property—phenotypic integration, which is characterized by stress-induced change of one trait leading to the corresponding changes of other traits through genetic, developmental, and functional connections [20, 21].

**Figure 1:**
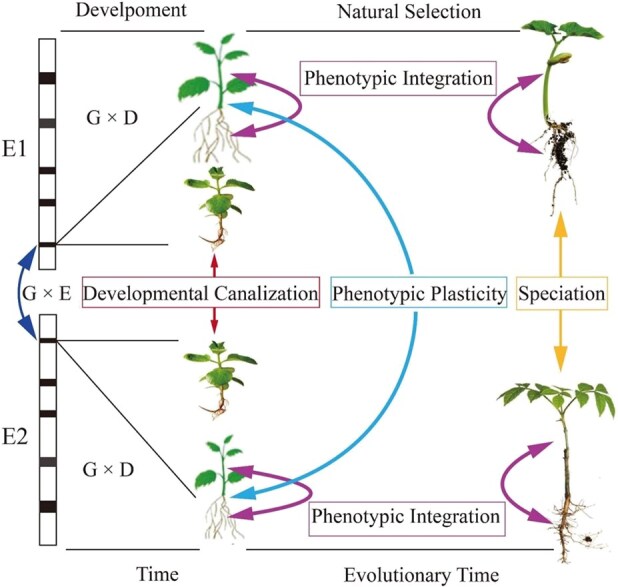
An eco-evo-devo model framework. The same gene on a chromosome exerts different genetic effects on the seedlings grown in two contrast environments (E1 and E2). The genetic effect interacts with development (G × D) to shape seedling traits at a late stage through three biological processes, developmental canalization (stress-invariant developmental change), phenotypic plasticity (stress dependent developmental change), and phenotypic integration (multi-trait developmental correlation change due to stress). Under natural selection, the traits displaying phenotypic plasticity diverge to morphologically, functionally and developmentally different phenotypes, possibly giving rise to speciation.

Figure 1 illustrates how each of the three eco-evo-devo properties exerts distinct impacts on stress response. Developmental canalization and phenotypic plasticity operate in a way that counteracts each other, because the former acts at a cost of the latter and vice versa. Despite such inconsistency, these properties are ultimately balanced, giving rise to overall stable phenotypes under stress. To that end, any stress response can be quantitatively measured as the difference between trait growth under stress-free and stress-exposed conditions [4]. This measure can reflect temporal changes of stress response due to interactions between an organism’s genes, environment, and development that maintain, release, or create phenotypic variation in stress-related traits. Through natural selection, this variation can be subsequently fixed, evolving novel phenotypes for stress adaptation (Fig. 1) [22].

 In this framework, the same gene on a chromosome exerts different genetic effects on the seedlings grown in two contrast environments (E1 and E2). The genetic effect interacts with development (G × D) to shape seedling traits at a late stage through three biological processes, developmental canalization (stress-invariant developmental change), phenotypic plasticity (stress-dependent developmental change), and phenotypic integration (multitrait developmental correlation change due to stress). Under natural selection, the traits displaying phenotypic plasticity diverge to morphologically, functionally, and developmentally different phenotypes, possibly giving rise to speciation.

In this mini-review article, we propose a new theoretical framework for systematically mapping the genetic control mechanisms of stress response using commonly used mapping and association populations. Different from traditional analytical methods, this framework introduces eco-evo-devo theory to define stress response as a dynamic curve of phenotypic differences and map this curve into bidirectional, signed, and weighted genetic interactome networks through the seamless integration of elements from different disciplines. This framework is defined as the functional game-graph theory (FunGG).

## Functional mapping and evolutionary game theory

As defined above, stress response is the developmental difference in growth under stress from stress-free control, which is mathematically a composite dynamic complex trait. Thus, understanding the genetic architecture of stress response is equivalent to mapping the composite dynamic trait. Wu and his team developed a statistical model, called functional mapping, which characterizes the developmental genetic control of dynamic traits [23–25]. Functional mapping has been leveraged to map a composite dynamic trait as a mathematical function of two or more dynamic traits [26]. This so-called composite functional mapping can be readily implemented to estimate and test the temporal pattern of genetic variation in stress response [27]. Although this approach can map pairwise epistatic effects due to two different loci, its derivation rationale was based on the marginal model, thus failing to perform a systematic characterization of genetic interactions among all loci. Also, the detection of epistatic effects by a marginal model requires an extremely large sample size (say 5000) [28], which is hardly met by the majority of genetic mapping studies.

The above issues have been overcome by integrating genetic interactions through the lens of evolutionary game theory [29]. According to classic game theory [30], a player (e.g. a gene) strives to maximize its payoff (i.e. the effect exerted by a gene) not only by optimizing its own strategy but also by considering strategies of its partners. Different players (i.e. genes in our case) keep adjusting their strategic decision rationally until the Nash equilibrium is reached [31]. Maynard Smith and Price leveraged game theory to evolutionary game theory [32], in which evolutionarily stable strategy (ESS) refines the equilibrium concept of the Nash equilibrium. The equilibrium of ESS does not rely on the rational assumption, but rather what can be approached through nature selection. Sun *et al.* [29] integrated evolutionary game theory and predator–prey theory [33] to build up a group of generalized Lotka–Volterra ordinary differential equations (gLVODE) that consider all genes as a system.

For each gene, gLVODE decomposes its observed genetic variance trajectory (by composite functional mapping) into its temporal independent component (due to this gene’s decisive strategy) and temporal dependent component (due to the decisive strategy of other genes). The independent genetic variance component arises when this gene is assumed to be in a socially isolated condition, whose magnitude implicates the intrinsic capacity of this gene, whereas the dependent genetic variance component results from (positive or negative) regulation for this gene by other genes, with its magnitude and sign reflecting the strength of gene–gene interaction and its causality. Sun *et al.* [29] implemented a nonparametric approach to smoothen temporal independent and dependent components, providing a procedure for estimating the values of these components and drawing their time-varying curves.

## Functional game-graph theory

The independent genetic variance components of different genes are coded as nodes and the dependent genetic variance components of different gene pairs as edges into mathematical graphs in which the roadmap of how each gene acts and how each pair of genes interacts can be charted at an unprecedented resolution of detail. The combination of functional mapping and evolutionary game theory through predator–prey equations into the graph paradigm forms a new theory—FunGG. Compared with traditional network approaches, FunGG is characterized by many unique advantages, as detailed in the subsequent sections.

### Mechanistic epistatic networks

**Figure 2:**
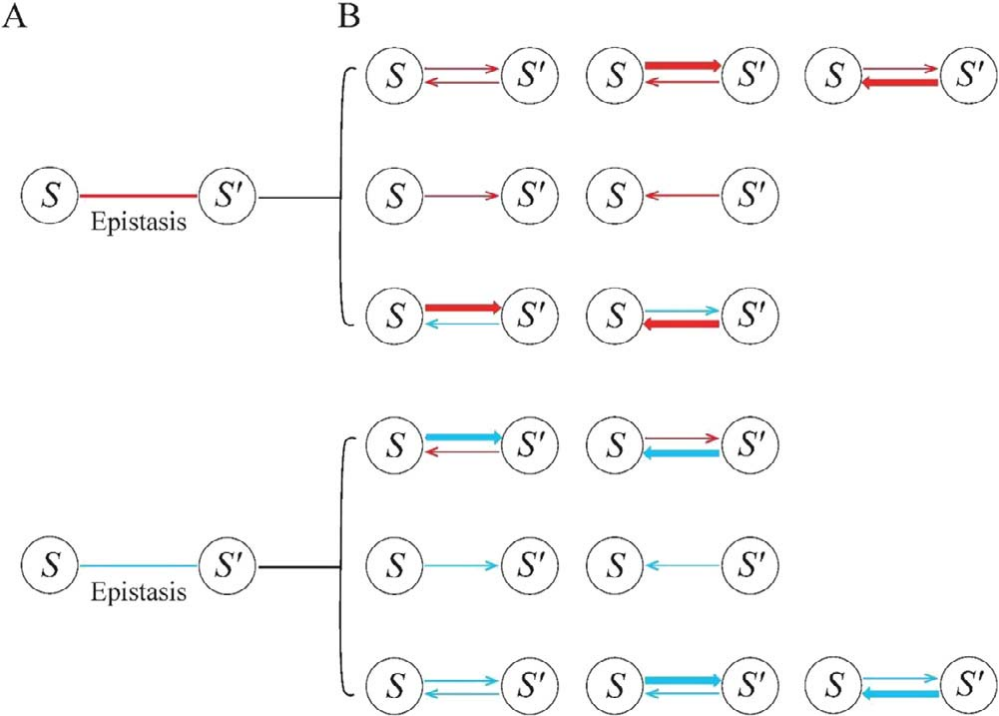
Epistasis observed between gene gene 𝑠 and 𝑠′ by a conventional approach (non-arrowed red and blue lines represent positive and negative interactions, respectively) (A), which can be attributed to different types of gene-gene interactions (B). Each of these types, which provides a precise characterization of epistatic formation, can be detected by FunGG. Arrowed lines stand for the causality of epistasis, with the thickness of lines proportional to the strength of epistasis. Red and blue lines represent promotion and inhibition, respectively.

FunGG can capture all topological properties of genetic interactions, which include strength, causality, sign, and feedback cycle. This is an improvement over existing quantitative genetic approaches that can only estimate the overall strength and sign of epistasis, but fail to characterize causality and feedback cycle. FunGG can also reveal the mechanistic underpinnings of the epistasis detected by traditional approaches (Fig. 2). In FunGG, positive (or negative) epistasis can be classified into three types: (i) the two genes promote (or inhibit) each other to produce synergistic (or antagonistic) epistasis, (ii) one gene promotes (or inhibits) the second, but the second has no effect on the first, to produce commensalistic or amensalistic epistasis, and (iii) although one gene inhibits (or promotes) the second, the latter promotes (or inhibits) the former, to produce aggressive or altruistic epistasis. FunGG can characterize the bidirectionality, sign, and extent of epistasis, significantly leveraging this phenomenon from mere phenomenological observation to mechanistic dissection.

### Dynamic epistatic networks

**Figure 3:**
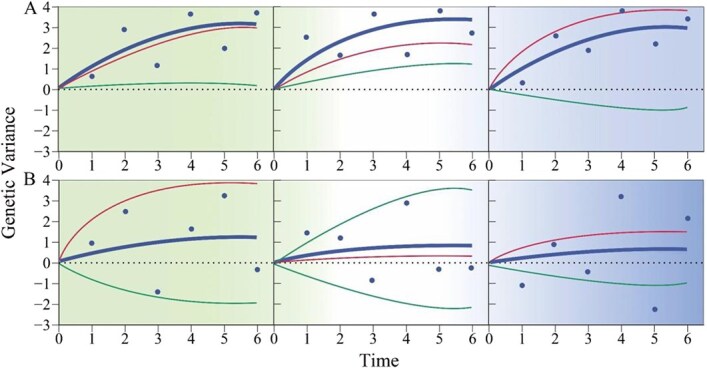
The dissection of genetic variation for stress response by FunGG. Temporal genetic variances of a stress-related trait (thick dark blue line) explained by significant genes (A) and insignificant genes (B) tested by conventional marginally based statistical approaches include two components, temporal independent genetic variances (thin red line) and temporal dependent genetic variances (thin green line) due to regulation by other genes. Dark blue dots denote estimated genetic variances at different time points, fitted by FunGG equations.

Stress response changes dynamically over time; thus, genetic networks that mediate it should also dynamically change. Traditional approaches can only reconstruct an overall genetic network from temporal data measured at a large number of time points. By estimating the values of independent and dependent components at each time point, FunGG reconstructs time-specific genetic networks, thus allowing gene–gene epistasis to be traced and visualized across temporal gradients. Using such temporal genetic networks, one can use FunGG to compare and test topological differences of genetic interactions among key time points, such as the timing of stress sensing, the emergence of stress resistance, and adaptation. This information is of fundamental importance to understand the developmental genetic mechanisms of stress response (Fig. 3).

### Topologically coordinated epistatic networks

Genetic networks reconstructed using FunGG are topologically coordinated in space, because they meet three basic properties of networks, causality, sparsity, and stability. As discussed above, FunGG is powerful for unraveling the causality of genetic interactions. In a gene community, each gene may possibly link with every other gene, but this fully connected network may not occur because they are vulnerable to random perturbations. FunGG implements variable selection, such as LASSO [34] and its variants [35, 36], to choose a small set of the most significant genes that link with a given gene, which implies that each is only linked with a small number of other genes. This further implies that sparsely connected genetic networks with strong robustness are reconstructed to buffer against any stochastic errors. By formulating and maximizing the likelihood of all genetic effect data, FunGG implements mathematical and statistical algorithms to obtain the optimal estimates of independent and dependent components. The networks reconstructed from these estimates under the optimality condition possess inherent stability.

### Multilayer, multiplex, multiscale, multifunctional networks

FunGG can also handle multidimensional genetic data. With the advent of advanced biotechniques, an increasing amount of genetic data for a complex trait can be collected and analyzed with ease. Reconstructing omnigenic interactome networks from these data is statistically challenging, given the fact that the number of genetic interactions increases exponentially with the increasing number of genes. This issue can be circumvented by introducing developmental modularity theory [37], which suggests that genes are more tightly linked with each other within the same modules than with genes from different modules [38, 39]. Each module represents a distinct subnetwork that may be sparsely linked with other communities. FunGG implements functional clustering [40, 41] to classify all genes into distinct modules based on their similarity of temporal genetic effects on stress response. Such classification of genes is equivalent to breaking down the omnigenic network into subnetworks at a small scale that allows us to trace, visualize, and interrogate the fine-grained roadmap of how each gene acts and interacts with every other gene to mediate stress tolerance, resistance, or sensitivity.

## Stress networks vs. genome-wide by environment interaction networks for stress response

The genetic studies of stress response require genotypically identical mapping populations to be reared in stress-free and stress-exposed environments. Such multienvironment mapping data can be used to dissect the phenotypic variation of a stress-related growth trait into different components due to the genetic effect, environmental effect, and genotype-environment interaction (GEI) effect at individual genes by a longitudinal two-way analysis of variance.

The genetic effect of a gene describes its overall effect on the mean of the growth trait expressed in different environments, whereas the environmental effect characterizes how much the population mean of growth varies from one environment to the next. These two types of effects provide limited information for characterizing the genetic variation of stress response as a basis for molecular breeding. The third type of effect, GEI, typically can unravel the genetic variation of growth response to stressful environments. While most existing approaches identify GEI effects due to individual genes, Wang *et al.* [42] developed a genome-wide by environment interaction study (GWEIS) model for coalescing all genes into interactome networks. GWEIS relaxes the linearity assumption of GEI effects by conventional quantitative genetic models; instead, it characterizes any (linear or nonlinear) form of how genes adjust their effects in response to environmental change.

Like stress networks, GWEIS networks are also methodologically built on the integration of functional mapping, evolutionary game theory, and predator–prey theory—the foundation of FunGG. However, the stress and GWEIS network models are different in several aspects of mapping gene-stress response relationships. Stress networks attempt to unveil how the topology of genetic networks mediate stress response, whereas GWEIS networks characterize how stress induces the topological change of genetic networks from stress-free to stress-exposed environments and vice versa. Stress networks investigate the genetic basis of how a growth trait responds to a stressful environment. This information is critical for evolutionary geneticists to infer the evolution of stress response. On the other hand, GWEIS networks characterize the environment-induced change of genetic control over the trait, a body of information of fundamental importance for breeders to select stress-adaptive genotypes. Taken together, stress networks can illustrate the role of genetic architecture in altering stress-relevant phenotypes, whereas GWEIS networks work better in uncovering the impact of stress on the genetic architecture of traits.

## A proof of concept: data structure, stress network reconstruction, and interpretation

**Figure 4:**
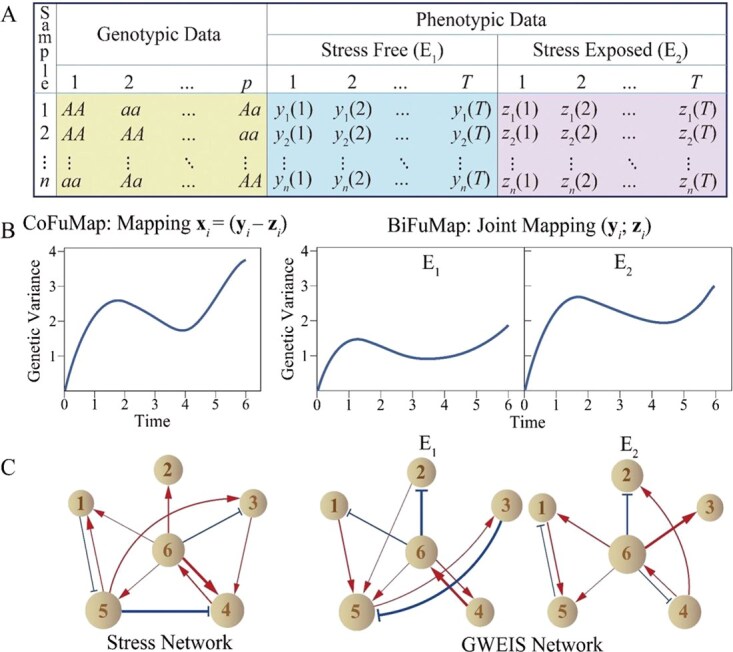
A diagram demonstrating the procedure of network reconstruction and its interpretations. (A) data structure of a mapping population involving genotypic data and phenotypic data collected in two contrasting environments. (B) Functional mapping of two-environment phenotypic traits. (C) Network reconstruction for phenotypic plasticity and GEIs.

Consider an SNP-genotyped (single nucleotide polymorphism, a DNA sequence variant caused by a change in a single nucleotide) mapping or association population of size *n* reared in stress-free (E1) and stress-exposed environments (E2). This ecological genetic experiment quantifies a stress-related phenotypic trait at T successive time points in two contrasting environments, with measurements initiated at the onset of stress. The corresponding diagram depicts the resulting data structure, comprising genotypic data at p SNPs and longitudinal phenotypic recorded in both environments. (Fig. 4A).

Let *g_j_*(*t*) denote the genetic variance of the trait at time *t* (*t* = 1, . . ., *T*) for SNP *j* (*j* = 1, . . ., *p*) estimated by composite functional mapping (CoFunMap) or bivariate functional mapping (BiFunMap) (Fig. 4B). Based on the estimated genetic variances, FunGG formulates a system of gLVODEs for individual SNPs, expressed as follows:


\begin{align*} dg_{i}(t)/dt&=Q_{\ j}(g_{i}(t);\Theta_{j})+\sum^{p}_{j^{\prime}=1}Q_{\ jj^{\prime}} (g_{\ j^{\prime}}(t);\Theta_{jj^{\prime}})\\&\quad+ e_{j}(t;\Psi_{j}), j^{\prime}\!\neq\! j\!=\!1, \ldots, p \end{align*}

where $(Q_{\ j}(g_{j}(t);\Theta_{j}) $ is the time-varying independent genetic variance component of SNP *j* that occurs when this SNP is assumed to be in isolation, $(Q_{\ jj^{\prime}} (g_{j^{\prime}} (t);\Theta_{jj^{\prime}}) $ is the time-varying independent genetic variance component of SNP *j* that results from the influence of SNP 𝑗′ on this SNP, and *e*_𝑗_(𝑡; $\Psi_{j} $) is the residual error distributed as (0, Σ*_j_*). The changes of independent and dependent genetic variances over time can be fitted by a nonparametric approach, specified by unknown parameters Θ_𝑗_ and Θ_𝑗𝑗′_, respectively. The error term is fitted by a structured antedependence model, specified by parameters Ψ_𝑗_ [43, 44]. For each SNP *j* as a prey, we implement LASSO-based variable selection to choose a small set of the most significant SNPs that behave as a predator to control the prey. Thus, fully connected gLVODEs described above are reduced to sparsely connected gLVODEs, which is the mathematical formulation of FunGG.

By solving the reduced gLVODEs, we reconstruct genetic interaction networks by coding the independent genetic variances as nodes and the dependent genetic variances as edges into graphs. Genetic variances by CoFunMap are used to infer stress networks, whereas those by BiFunMap are used to infer GWEIS networks (Fig. 4C). The hypothesized networks include six SNPs, where nodes are denoted by circles with sizes proportional to the component values and edges are denoted by arrowed red lines (up-regulated epistasis) and T-shaped blue lines (down-regulated epistasis), with thickness proportional to the strength of epistasis. Based on the above procedure, we designed a genetic mapping experiment using a full-sib family of two Euphrates poplar trees, a desert-adapted tree species, sampled from a natural population. The family was cloned and cultured in salt-exposed and salt-free conditions, allowing the phenotypic plasticity of a stress response-related trait to be calculated based on the difference of trait values between the two conditions. FunGG reconstructed a multilayer stress network from genome-wide SNP genotypes for the growth response of Euphrates poplar to salt stress [27]. This network identifies the marked effect of gene *RPL38A* (encoding a structural constituent of ribosomes) on saline response that is due to up-regulation by protein transport-encoding *LOC105121577*. The same study population was also analyzed by the GWEIS network model, finding that GEI at a gene may be modulated by other regulators [42]. In both stress networks and GWEIS networks, the majority of genetic interactions identified by FunGG can be annotated by characterized protein–protein interactions.

## Concluding remarks

Stress response is an important biological phenomenon involving complex genetic architecture [45, 46]. Forward genetic approaches have been instrumental for identifying key genes or biochemical pathways involved in stress adaptation, but seem to be limited in charting a complete picture of genetic control over stress response. Reverse genetic strategies based on genetic mapping and association studies can overcome this limitation, but their application solely relies on the new concept, theory, and method that can assemble a full set of genes into interaction networks.

In this article, we describe how FunGG can trace and visualize the detailed journey through which each and every gene streamlines its effect on stress response, either directly or indirectly. It can predict whether the significance of a gene detected by conventional reductionist approaches is due to its own intrinsic capacity or favorable up-regulation by other genes, or both. FunGG can also infer whether an insignificant gene tested is insignificant because of its own poor capacity or because it is down-regulated by negative regulators. The FunGG proposed in this article is derived from longitudinal data of stress response. It has now been extended to infer stress networks from static data by integrating allometric scaling law [47, 48]. The statistical behavior of FunGG has been investigated and validated experimentally or through computer simulation in previous studies [27, 42].

The fine-grained roadmaps of genetic effects recorded in stress networks can potentially inform geneticists to effectively perform marker-assisted selection or gene editing for improving stress-adaptive traits. By silencing their negative regulators, those seemingly insignificant genes in a socialized condition can be activated to release their effects on stress response. In theory, this strategy can be used as a generic approach to interpret and retrieve missing heritability [42]. While there has been a rich literature on gene regulatory networks inferred from omics data [49, 50], our FunGG is a brave attempt to reconstruct genetic interactome networks for complex traits from genotype data. The future integration of gene regulatory networks into genetic interactome networks can systematically characterize all genes and their epistasis. Such integration would potentially prove a paradigm shift for stress genetic research from a long-predominant reductionist thinking to a more mechanistically driven holistic, systems-oriented philosophy.

## References

[1] Zhu JK . Abiotic stress signaling and responses in plants. Cell. 2016;167:313–24 27716505 10.1016/j.cell.2016.08.029 PMC5104190

[2] Himanen SV, Sistonen L. New insights into transcriptional reprogramming during cellular stress. J Cell Sci. 2019;132:jcs238402 31676663 10.1242/jcs.238402

[3] Bechtold U, Field B. Molecular mechanisms controlling plant growth during abiotic stress. J Exp Bot. 2018;69:2753–8 29788471 10.1093/jxb/ery157 PMC5961130

[4] Zhang H, Zhao Y, Zhu JK. Thriving under stress: how plants balance growth and the stress response. Dev Cell. 2020;55:529–43 33290694 10.1016/j.devcel.2020.10.012

[5] Gupta A, Rico-Medina A, Caño-Delgado AI. The physiology of plant responses to drought. Science. 2020;368:266–9 32299946 10.1126/science.aaz7614

[6] Costa-Mattioli M, Walter P. The integrated stress response: from mechanism to disease. Science. 2020;368:eaat5314 32327570 10.1126/science.aat5314 PMC8997189

[7] Terhorst A, Sandikci A, Keller A. et al. The environmental stress response causes ribosome loss in aneuploid yeast cells. Proc Natl Acad Sci U S A. 2020;117:17031–40 32632008 10.1073/pnas.2005648117 PMC7382292

[8] Nolan TM, Vukašinović N, Liu D. et al. Brassinosteroids: multidimensional regulators of plant growth, development, and stress responses. Plant Cell. 2020;32:295–318 31776234 10.1105/tpc.19.00335 PMC7008487

[9] Zhang H, Zhu J, Gong Z. et al. Abiotic stress responses in plants. Nat Rev Genet. 2022;23:104–19 34561623 10.1038/s41576-021-00413-0

[10] Waddington CH . The Strategy of the Genes. London: George Allen & Unwin Ltd; 1957:

[11] Bradshaw AD . Evolutionary significance of phenotypic plasticity. Adv Genet. 1965;13:115–55

[12] Sultan SE . Phenotypic plasticity for plant development, function and life history. Trends Plant Sci. 2000;5:537–42 11120476 10.1016/s1360-1385(00)01797-0

[13] Pigliucci M . Evolution of phenotypic plasticity: where are we going now? Trends Ecol Evol. 2005;20:481–6 16701424 10.1016/j.tree.2005.06.001

[14] Fusco G, Minelli A. Phenotypic plasticity in development and evolution: facts and concepts. Introduction. Philos Trans R Soc Lond B Biol Sci. 2010;365:547–56 20083631 10.1098/rstb.2009.0267 PMC2817147

[15] Wang Z, Pang X, Wu W. et al. Modeling phenotypic plasticity in growth trajectories: a statistical framework. Evolution. 2014;68:81–91 24111588 10.1111/evo.12263

[16] Yang D, Jin Y, He X. et al. Inferring multilayer interactome networks shaping phenotypic plasticity and evolution. Nat Commun. 2021;12:5304 34489412 10.1038/s41467-021-25086-5 PMC8421358

[17] Ratikainen II, Kokko H. The coevolution of lifespan and reversible plasticity. Nat Commun. 2019;10:538 30710077 10.1038/s41467-019-08502-9 PMC6358619

[18] Vaidya AS, Helander JDM, Peterson FC. et al. Dynamic control of plant water use using designed ABA receptor agonists. Science. 2019;366:eaaw8848 31649167 10.1126/science.aaw8848

[19] Pigliucci M, Preston K. Phenotypic Integration: Studying the Ecology and Evolution of Complex Phenotypes. Oxford; New York: Oxford University Press; 2004:

[20] Schlichting CD, Pigliucci M. Phenotypic Evolution: A Reaction Norm Perspective. Sunderland, MA: Sinauer Associates Inc; 1998:

[21] Abouheif E, Favé MJ, Ibarrarán-Viniegra AS. et al. Eco-evo-devo: the time has come. Adv Exp Med Biol. 2014;781:107–25 24277297 10.1007/978-94-007-7347-9_6

[22] Gilbert SF, Bosch TCG, Ledón-Rettig C. Eco-Evo-Devo: developmental symbiosis and developmental plasticity as evolutionary agents. Nat Rev Genet. 2015;16:611–22 26370902 10.1038/nrg3982

[23] Ma CX, Casella G, Wu R. Functional mapping of quantitative trait loci underlying the character process: a theoretical framework. Genetics. 2002;161:1751–62 12196415 10.1093/genetics/161.4.1751 PMC1462199

[24] Wu RL, Lin M. Functional mapping—how to map and study the genetic architecture of dynamic complex traits. Nat Rev Genet. 2006;7:229–37 16485021 10.1038/nrg1804

[25] Sun LD, Wu RL. Mapping complex traits as a dynamic system. Phys Life Rev. 2015;13:155–85 25772476 10.1016/j.plrev.2015.02.007 PMC5467743

[26] Sang MM, Shi H, Wei K. et al. A dissection model for mapping complex traits. Plant J. 2019;97:1168–82 30536697 10.1111/tpj.14185

[27] Feng L, Dong T, Jiang P. et al. An eco-evo-devo genetic network model of stress response. Hortic. Res. 2022;9:uhac135 36061617 10.1093/hr/uhac135 PMC9433980

[28] Zuk O, Hechter E, Sunyaev SR. et al. The mystery of missing heritability: genetic interactions create phantom heritability. Proc Natl Acad Sci U S A. 2012;109:1193–8 22223662 10.1073/pnas.1119675109 PMC3268279

[29] Sun L, Dong A, Griffin C. et al. Statistical mechanics of clock gene networks underlying circadian rhythms. Appl Phys Rev. 2021;8:021313

[30] von Neumann J, Morgenstern O. Theory of Games and Economic Behavior. Princeton, NJ: Princeton University Press; 1944.

[31] Nash JF . Equilibrium points in N-person games. Proc Natl Acad Sci U S A. 1950;36:48–9 16588946 10.1073/pnas.36.1.48 PMC1063129

[32] Maynard Smith JM, Price GR. The logic of animal conflict. Nature. 1973;246:15–8

[33] Abrams PA . The evolution of predator-prey interactions: theory and evidence. Annu Rev Ecol Syst. 2000;31:79–105

[34] Tibshirani R . Regression shrinkage and selection via the lasso. J Roy Stat Soc Ser B (Method). 1996;58:267–88

[35] Zou H . The adaptive lasso and its oracle properties. J Am Stat Assoc. 2006;101:1418–29

[36] Wang H, Leng C. A note on adaptive group lasso. Comput Stat Data Anal. 2008;52:5277–86

[37] Wagner GP, Pavlicev M, Cheverud JM. The road to modularity. Nat Rev Genet. 2007;8:921–31 18007649 10.1038/nrg2267

[38] Espinosa-Soto C . On the role of sparseness in the evolution of modularity in gene regulatory networks. PLoS Comput Biol. 2018;14:e1006172 29775459 10.1371/journal.pcbi.1006172 PMC5979046

[39] Mäkinen H, Sävilammi T, Papakostas S. et al. Modularity facilitates flexible tuning of plastic and evolutionary gene expression responses during early divergence. Genome Biol Evol. 2018;10:77–93 29293993 10.1093/gbe/evx278 PMC5758911

[40] Kim BR, Zhang L, Berg A. et al. A computational approach to the functional clustering of periodic gene-expression profiles. Genetics. 2008;180:821–34 18780724 10.1534/genetics.108.093690 PMC2567383

[41] Wang Y, Xu M, Wang Z. et al. How to cluster gene expression dynamics in response to environmental signals. Brief Bioinform. 2012;13:162–74 21746694 10.1093/bib/bbr032 PMC3294239

[42] Wang H, Ye M, Fu Y. et al. Modeling genome-wide by environment interactions through omnigenic interactome networks. Cell Rep. 2021;35:109114 33979624 10.1016/j.celrep.2021.109114

[43] Zhao W, Chen YQ, Casella G. et al. A non-stationary model for functional mapping of complex traits. Bioinformatics. 2005;21:2469–77 15769837 10.1093/bioinformatics/bti382

[44] Zhao W, Hou W, Littell RC. et al. Structured antedependence models for functional mapping of multiple longitudinal traits. Stat Appl Genet Mol Biol. 2005;4:33 10.2202/1544-6115.1136 16646852

[45] Rigal A, Doyle SM, Ritter A. et al. A network of stress-related genes regulates hypocotyl elongation downstream of selective auxin perception. Plant Physiol. 2021;187:430–45 34618142 10.1093/plphys/kiab269 PMC8418399

[46] Vakulenko S, Grigoriev D. Deep gene networks and response to stress. Mathematics. 2021;9:3028

[47] Griffin C, Jiang L, Wu R. Analysis of quasi-dynamic ordinary differential equations and the quasi-dynamic replicator. Physica A Stat Mech Appl (Physica A). 2020;555:124422

[48] Wu RL, Jiang L. Recovering dynamic networks in big static datasets. Phys Rep. 2021;912:1–57

[49] Badia-i-Mompel P, Wessels L, Müller-Dott S. et al. Gene regulatory network inference in the era of single-cell multi-omics. Nat Rev Genet. 2023;24:739–54 37365273 10.1038/s41576-023-00618-5

[50] Jain M . Gene regulatory networks in abiotic stress responses via single-cell sequencing and spatial technologies: advances and opportunities. Curr Opin Plant Biol. 2024;82:102662 39541907 10.1016/j.pbi.2024.102662

